# Nature of charge transport and *p*-electron ferromagnetism in nitrogen-doped ZrO_2_: An *ab initio* perspective

**DOI:** 10.1038/srep08586

**Published:** 2015-02-26

**Authors:** Huanfeng Zhu, Jing Li, Kun Chen, Xinyu Yi, Shuai Cheng, Fuxi Gan

**Affiliations:** 1 Department of Optical Science and Engineering, Fudan University, Shanghai 200433, China; 2 Shanghai Engineering Research Center of Ultra-Precision Optical Manufacturing, Fudan University, Shanghai 200433, China; 3 Key Laboratory of Micro and Nano Photonic Structure (Ministry of Education), Fudan University, Shanghai 200433, China

## Abstract

Zirconium dioxide provides an exceptional prototype material for studying the redistribution of the polaron holes and its magnetic coupling with their nearby anions owning to the difference oxygen binding behavior in the monoclinic phase. Here, we perform a comprehensive study of the *p*-electron magnetism in the nitrogen doped 2 × 2 × 2 monoclinic ZrO_2_ based on spin-polarized density functional theory. Nitrogen substitutions make the system display half-metallic properties, and the origin of room temperature ferromagnetism ascribes to the *p*-*p* coupling interaction between N 2*p* and the host 2*p* states. The charge density difference and Mülliken population analyses provide evidences of charge redistributions. Our results reveal that the polaron transfer may alter the magnetic properties and it is greatly facilitated ferromagnetic coupling if the polaron holes are localized around a single anion dopant.

The defect-induced magnetism involving only *s*- and *p*-electron elements have captured great interest and exhibited promising potential applications in spintronics devices. Doping 2*p* light elements (LE) into oxide semiconductors, what is known as a model system of diluted magnetic semiconductors (DMSs)^1^,^2^,^3^,^4^, the room temperature ferromagnetism (RTFM) can be achieved. For instance, RTFM was reported in C- or N-doped ZnO^5^,^6^,^7^,^8^, TiO_2_^9^,^10^,^11^ thin films and Li-doped ZnO^12^ nanorods. Theoretically, possible ferromagnetism (FM) was also predicted in C-, or N-doped TiO_2_^13^,^14^, ZnO^15^,^16^, CaO^17^,^18^ and MgO^19^,^20^,^21^,^22^ by density functional theory (DFT) or self-interaction correction (SIC) calculations. Compared to the conventional 3*d* transition-metal (TM)-doped DMSs^23^,^24^,^25^,^26^,^27^, LE-doped DMSs can provide a novel way to exclude the problem of clusters or secondary phases formed by doping 3*d* TM elements. Moreover, the spatially extended *p* states of the host and the impurities may able to extend the magnetic interaction to longer range. Various experimental and theoretical explorations have shown that the exchange interactions between the structural defects are believed to be responsible in establishing the magnetic moment. However, it remains obstacle that the relationship between valence band holes and origin of stable magnetic ordering in these materials. Therefore, a comprehensive understanding on the *p*-electron magnetism is essential for further exploring other potential DMSs.

The reasons we choose zirconium dioxide (ZrO_2_) as a prototypical DMS are as follows. (i) ZrO_2_ as a promising candidate for gate dielectric material^28^,^29^,^30^,^31^,^32^, has attracted much attention due to its relatively large dielectric constant (~25), wide band gap (5.1–7.8 eV), high breakdown field, and good thermal stability. Introducing ferromagnetism into the nonmagnetic (NM) ZrO_2_ may open a new avenue for its applications. (ii) Due to difference oxygen binding behaviors in monoclinic ZrO_2_ (*m*-ZrO_2_), it is an exceptional prototype material for understanding of the relationship between electronic structure and the *p*-electron characters in these DMSs. Inspired by the prediction of Ostanin *et al*.^33^ that cubic ZrO_2_ should have a high Curie temperature (<500 K) by doping Mn, lots of efforts have focused on Mn-doped cubic ZrO_2_ but rare attentions were paid to LE-doped *m*-ZrO_2_, especially for doping N^34^,^35^,^36^.

Unlike TiO_2_、 CeO_2_ and SnO_2_, oxygen sites in the *m*-ZrO_2_ can be divided into two types according to their binding behavior, one is bonded with three Zr atoms (labeled as O3) and the other is bonded with four Zr atoms (labeled as O4) [see Figs. 1(a) and 1(b)]. The projected density of states (PDOS) of the 2*p* orbitals for the O3 and O4 are presented in Fig. 1(d). At the first glance, the 2*p* orbitals of O3 can accommodate more holes than that in the 2*p* orbitals of O4 near the Fermi level. It implies that the O4 2*p* shell should be first fully occupied compare to the O3 2*p* shell while sufficient holes are injected. It can be anticipated that the redistribution of the induced holes and the interaction with their nearby atoms will have a significant impact on establishing the RTFM.

## Results

### Structure stability

Let us first consider a Zr_32_O_63_N supercell with a single N substituted at an O site, accompany a formation of a doped hole. After a structural relaxation, the N substituted at O3 site (N_O3_) model system is found to have a lower energy of 65.4 meV than that the N substituted at O4 site (N_O4_) model system. This should be due to the fact that N_O3_ has a more freedom to relax than the N_O4_. In addition, both spin-polarized and non-spin-polarized states are calculated to check the stability of the magnetization. The results reveal that both the model system of N_O3_ and that of N_O4_ favor the energy differences of about 387.5 and 293.1 meV between the spin-polarized state and the non-spin-polarized state respectively. The probability of incorporating a defect in a crystal is determined by the formation energy. The calculated formation energy of N_O3_ (or N_O4_) is 3.92 eV (or 3.99 eV) and −1.56 eV (or −1.49 eV) for the O-rich condition and Zr-rich condition, respectively.

### Electronic properties

The calculated total density of states (TDOS) and PDOS for the N 2*p*, the neighboring Zr 4*d* and O 2*p* states are shown in Fig. 2. It is found there exist significant hybridization between the N 2*p* states and O 2*p* states around the Fermi level (*E_F_*), suggesting a strong *p*-*p* coupling interaction between them. Such a strong *p*-*p* interaction makes the energy levels near the *E_F_* spin split. The partially filled spin-down band split off from the fully occupied spin-up band via Hund exchange, resulting typical FM half-metallic character, which is important for the spintronics applications^37^. Additionally, nitrogen substituting for oxygen introduces some hole states peaked abound 1.0 eV above *E_F_*, which are mainly originated from N 2*p*, O 2*p* and Zr 4*d* orbitals. The total magnetic moment of the supercell is 1.00 *μ*_B_ corresponding to the single excess *p* electron provided by the N dopant. This is consistent with the previous results of N-doped ZnO^15^, TiO_2_^14^, In_2_O_3_^38^. The moments are mainly localized on the N 2*p* orbital (~0.86 *μ*_B_), with minor contributions of the O (0.18 *μ*_B_) and the neighboring Zr atoms is polarized antiferromagnetically with a magnetic moment of −0.04 *μ*_B_ (See the Supplementary Information for more details).

The band structure calculations suggest that the localized holes induced by N dopant mediate the FM interaction. The carriers, i.e. holes, which were introduced by the substitution of N at O sites, reside not only on the N 2*p* orbitals but also on its near neighboring O atoms. The holes near each anion impurity tend to align parallel to the moment of the impurity ion under the *p*-*d* hybridization-like *p-p* coupling interaction^5^,^39^. Sufficient spin-polarized holes can mediate the spin alignment of the N dopant atoms, resulting in an indirect strong FM coupling among them. Therefore, the mechanism responsible for the FM coupling in the N-doped ZrO_2_ can be classified as the *p-p* coupling interaction between the impurity 2*p* states and the host 2*p* states.

### Magnetic coupling

**Figure 1:**
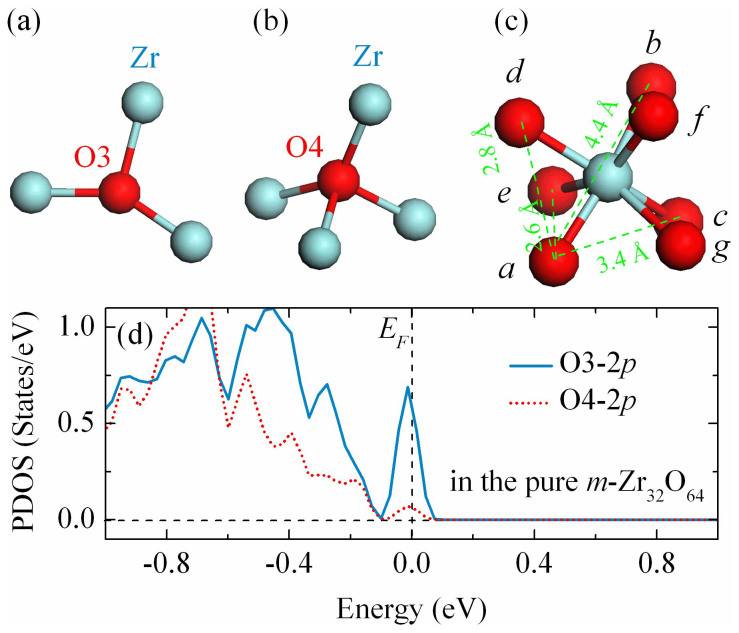
The geometries around (a) O3, (b) O4, and (c) Zr site in the *m*-ZrO_2_. The cyan and red balls represent Zr and O atoms, respectively. (d) The PDOS for the O3 and O4 in the 2 × 2 × 2 ZrO_2_ supercell. The Fermi level is set to zero.

**Figure 2:**
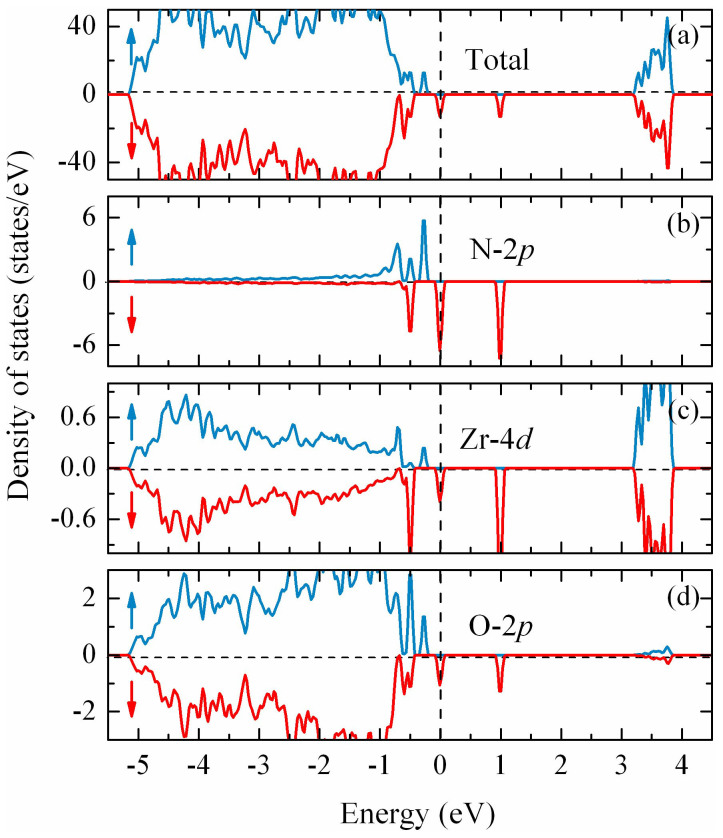
Calculated spin-resolved TDOS and PDOS of Zr_32_O_63_N with single N substituted at O3-type site. The TDOS shows in (a). The (b), (c) and (d) plot the PDOS of N 2*p* states, the neighboring Zr 4*d* states and the neighboring O 2*p* states, respectively. The positive (negative) values represent the spin-up (spin-down) states. The vertical dotted line indicates the Fermi level at 0 eV.

Next, we examine the magnetic coupling of these magnetic moments. To understand this issue, we calculate four nonequivalent structural configurations of Zr_32_O_62_N_2_, which are constructed by replacing two O atoms by N with various N-N distances. Here, the “dimer” of N-Zr-N configuration is only considered because we find the magnetic coupling to be negligible small when the N-N pair is well separated. The (*i*, *j*) configuration is adopted to denote an N-N pair, in which two O sites are replaced by N at *i* and *j* sites, as shown in Fig. 1(c). This amounts to the doping concentration of 3.13%. The magnetic coupling strength can be obtained from the energy difference between FM and AFM states (Δ*E*_FM-AFM_ = *E*_FM_-*E*_AFM_). A positive value means the FM state is favored and vice versa. Δ*E* is the relative energy with respect to the (*a, b*) configuration which has the lowest total energy. The results are summarized in Table 1. It is found that the Δ*E* becomes smaller when the N-N distance gets larger, and hence the doped N atoms do not have a tendency of clustering. It is noteworthy that the FM states always exhibit a lower energy than the AFM states except for the (*a*, *d*) configuration in which the N-N distance is 2.8 Å with the bond angle ∠N-Zr-N near 90°. It is well known that the Curie temperature in the mean field approximation (MFA) *T*_c_ is calculated as *k*_B_*T*_c_ = (2/3*c*)Δ*E*_FM-AFM_, where *k*_B_ is Boltzmann constant^35^,^40^,^41^. The MFA estimates *T*_c_ for DMSs with low concentrations within 5~10% errors. The *T*_c_ is always very lower than the room temperature, due to the short-range magnetic exchange interaction of deep impurity states in wide band-gap semiconductors, percolation of the ferromagnetic coupling is difficult to achieve for small concentrations. However, in the case of (*a*, *b*) configuration, the Δ*E*_FM-AFM_ value reaches 372.7 meV, larger that of N-doped TiO_2_^14^ and N-doped In_2_O_3_^38^ which are known to be RTFM. Therefore, such a strong ferromagnetic interaction implies that the RTFM for N-doped ZrO_2_ is quite feasible considering the simple estimate. Though not shown here, our additional similar calculations for Zr_32_O_62_N_2_ with a long N-N distance (7.4 Å) reveal that the FM and AFM state are degenerate, whereas the total energy is 412.3 meV higher compared to the ground state of (*a*, *b*) configuration (See the Supplementary Information for more details). For comparison, let's focus on the largest and shortest N-N distance in the “dimer” of N-Zr-N configuration, corresponding to the (*d*, *e*) configuration and the ground state of (*a*, *b*) configuration, respectively.

**Figure 3:**
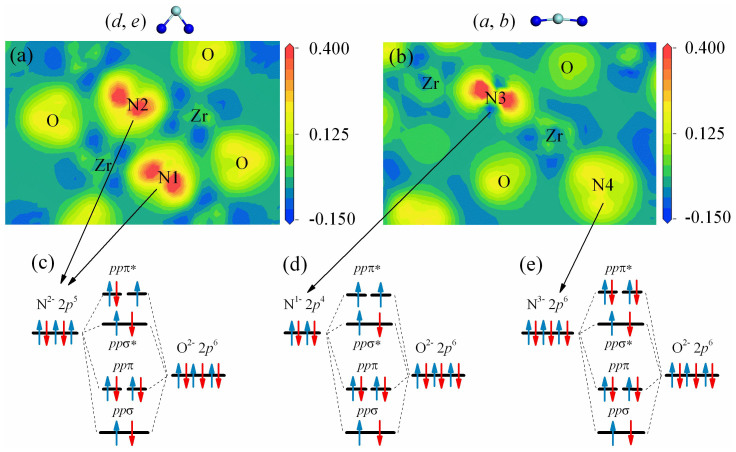
The charge density differences for the (*d*, *e*) and (*a*, *b*) configurations are shown in (a) and (b). The schematic energy level diagram of pair interaction between O^2−^ ions and N^2−^, N^1−^ and N^3−^ ions are illustrated in (c), (d) and (e), respectively.

**Figure 4:**
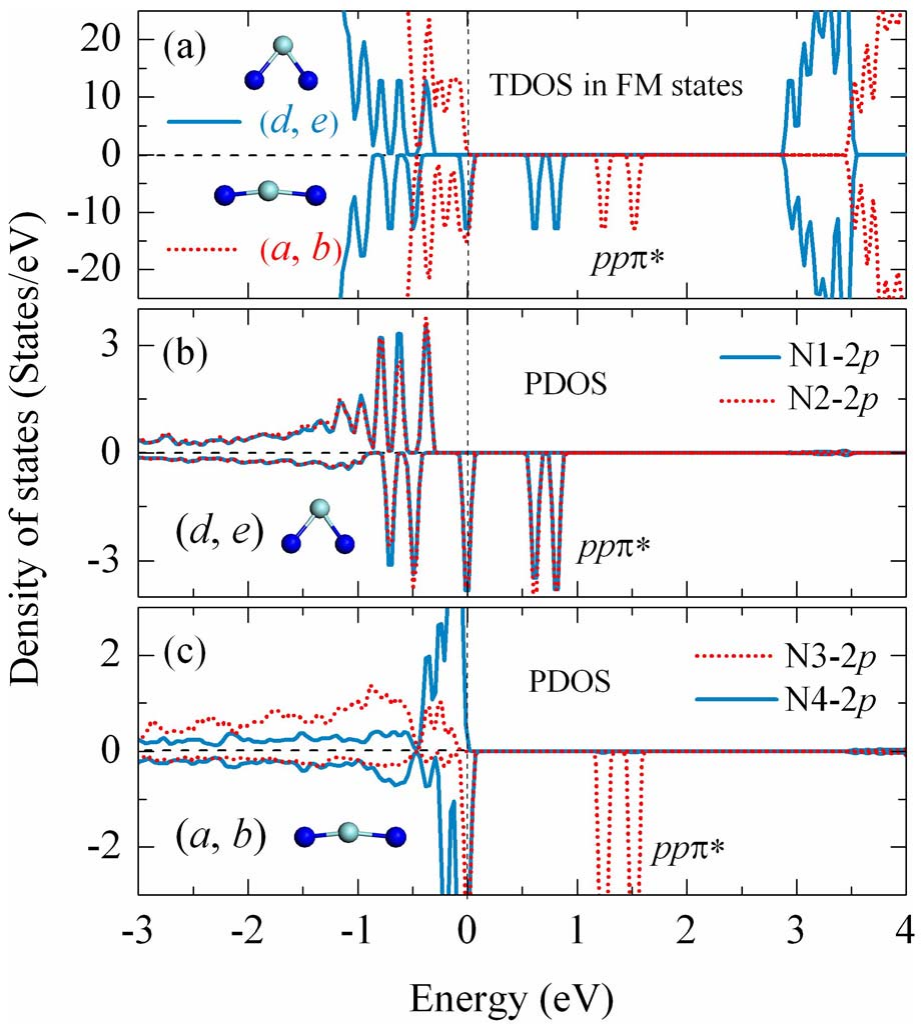
The spin-resolved TDOS and PDOS for the (*d*, *e*) and (*a*, *b*) configurations. (a) TDOS. The N 2*p* PDOS of (*d*, *e*) and (*a*, *b*) configurations in the FM states are illustrated in (b) and (c) respectively. The Fermi level is indicated by the dashed vertical line.

For a deeply understanding of the physical origin of the ferromagnetism, the charge density difference of (*a*, *b*) and (*d*, *e*) configurations in FM states is calculated, as shown in Figs. 3(a) and 3(b), respectively. A weak covalent bonding can be seen between doped N atoms and its nearest-neighboring O atoms, suggesting that there exists a coupling interaction. Furthermore, in (*d*, *e*) configuration, the two doped N atoms which both N1 and N2 substituted at O3-type sites gain almost equivalent electrons from their neighboring Zr atoms. However, the situation is quite different in (*a*, *b*) configuration. The N4, where N substitutes O at O4-type site, 2*p* shell is almost completely filled as compared to the N3 where N substitutes O at O3-type site, just like the fully filled O^2−^ 2*p* shell. These observations are consistent with the analysis in Fig. 1(d) that O4 2*p* shell is more easily to fully occupy than the O3 2*p* shell. This charge redistribution can also be quantitatively estimated by considering the Mülliken population analysis^42^. The charges of Zr, O and N atoms at different configurations are given in Table 2. To start with, we calculate the number of electrons on each atom in bulk ZrO_2_, which results in charge states of Zr and O atoms of +1.50*e* and −0.74*e*, respectively. Note that these values are far from the formal ones corresponding to the ideal ionic situation (Zr^4+^ and O^2−^), owning to the spatial division and the partial covalent feature of the Zr-O bonds. For (*d*, *e*) configuration, a Mülliken population analysis indicates that the local charges of N1 and N2 ions are −0.65*e* and −0.64*e*, whereas the neighboring of Zr and O remain essentially unchanged. Similar calculation for (*a*, *b*) configuration reveals that the N-N pair is strongly polarized, with charge states of −0.88*e* and −0.45*e* on the N4 and N3 sites, respectively. Given the above observations, the N1 (N2), N3 and N4 ions should have their electron configuration like N^2−^ (*s*^2^*p*^5^), N^1−^ (*s*^2^*p*^4^) and N^3−^ (*s*^2^*p*^6^), respectively. Due to the coupling interaction between N 2*p* and O 2*p*, *ppπ* and *ppσ* states are formed. The schematic energy level diagram of the *p*-*p* hybridization between O^2−^ and the above three kinds of N charge configurations (N^2−^, N^1−^, N^3−^) are illustrated in Figs. 3(c), 3(d) and 3(e). The nearly degenerate *ppπ* antibonding states are half-filled, 3/4 filled and fully filled, resulting 1.0 *μ*_B_, 2.0 *μ*_B_ and 0.0 *μ*_B_ total magnetic moment, consistent with the DOS in Figs. 4(a)–4(c).

**Figure 5:**
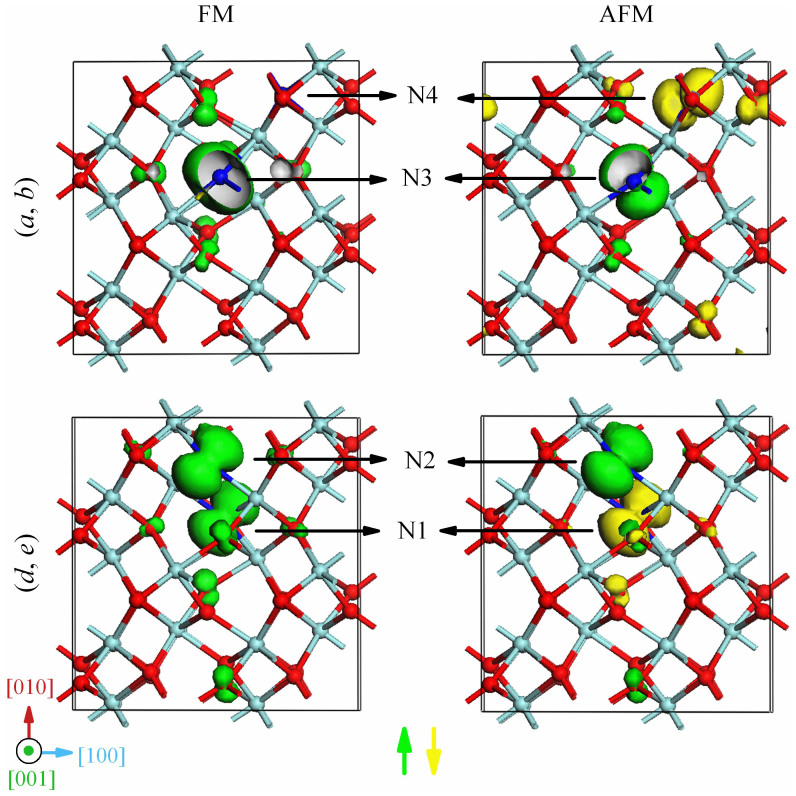
The isosurface SCD of (*a, b*) and (*d*, *e*) configurations in the FM states and the AFM states. The cyan, red and blue balls represent Zr, O and N atoms, respectively. The green (yellow) isosurface correspond to positive (negative) spin charge densities. The isovalue is set to 0.02 e/Å^3^.

In order to further understand the nature of magnetic coupling in Zr_32_O_62_N_2_, the spin charge density (SCD) distribution of (*a*, *b*) and (*d*, *e*) configurations in the FM states and the AFM states are shown in Fig. 5. As can be seen, in (*d*, *e*) configuration, the *p*-like shape spin charge resides mainly on both the two N impurities and they have identical spin direction in the FM states while opposite direction in the AFM states. It indicates that there existed a strong FM coupling interaction induced by the N 2*p* orbitals [see also Fig. 4(b)]. Meanwhile, the lobes of N 2*p* orbitals are towards the lobes of O 2*p* orbitals to maximize the overlaps, which manifests that the *p*-*p* interaction does extend to the neighboring O ions, similar to the case of single N doping. In great contrast, for (*a*, *b*) configuration in the FM states, the main features are that the spin charge is mostly contributed by the N4 dopant and N-N pair coupling interaction nearly vanished [see also Fig. 4(c)]. This can be understood by noticing that the N-N distance is 4.4 Å, almost double of the distance in (*d*, *e*) configuration. Such a long distance effectively degenerate the N-N pair coupling interaction.

## Discussion

Now we turn to address the question of what stabilizes the ferromagnetic ground state of (*a*, *b*) configuration. Spontaneous ferromagnetism can be evaluated according to the “Stoner Criterion”^43^,^44^: *D*(*E_F_*)*J* > 1, where *D*(*E_F_*) is the DOS at the Fermi level and *J* denotes the strength of the exchange interaction. The *D*(*E_F_*) is proportional to *m*^3/2^(*E*_VBM_-*E*_F_)^1/2^, where *m* is the effective mass and *E*_VBM_ is the energy of the valence band maximum (VBM). Due to the stronger N3-Zr than N4-Zr bonds in the (*a*, *b*) configuration as indicated by their bond length (N3-Zr~2.05 Å, N4-Zr~2.22 Å), the N3 spin-up states are occupied and the occupation of the spin-down states will decrease. Thus the spin charge tend to accumulate around the N3 atom, which makes the defect level deeper and wave function more localized, resulting in a larger *D*(*E_F_*), and a larger spin-exchange splitting *J* of the band, as can be seen in Figure 4(a). The enhanced exchange splitting stabilizes the FM state, it can cause the system to be ground state. These results therefore indicate that it will be easier to obtain stable ferromagnetism if the injected holes are not shared by several neighboring anion atoms, but localize around a single anion. A similar situation was observed in the different system of C-doped ZnO^45^.

In summary, our *ab initio* calculation results indicate that the substitutional N induces spin-split impurity states near the Fermi level and leads to the half-metallic property. The total magnetic moment is found to be proportional to the number of injected holes in the supercell. The magnetic moments in this system are attributed to the strong *p*-*p* coupling interaction between the localized N 2*p* states and the neighboring O 2*p* states. An analysis from charge density difference and Mülliken population analysis was developed to explain the charge transfer due to the different binding behavior of N substituted at O sites. Our findings pave the way for interpreting further the nature of *p*-electron magnetism in LE-doped oxide DMSs.

## Methods

*ab initio* calculations are performed by using density functional theory (DFT) with the plane-wave pseudopotential (PWP) approach as implemented in the Cambridge Sequential Total Energy Package (CASTEP)^46^. A generalized gradient approximation (GGA)^47^ in the form of Perdew-Burke-Ernzerhof (PBE) is used for the exchange correlation functional. The plane-wave cutoff energy is chosen to be 300 eV and a 2 × 2 × 2 Monkhorst-Pack *k*-point mesh is adopted for integrations over the Brillouin zone. The valence electron configurations for Zr, O and N are [Kr]4*d*^2^5*s*^2^, [He]2*s*^2^2*p*^4^ and [He]2*s*^2^2*p*^3^, respectively. For structural relaxation, all the atomic positions are fully optimized until the atomic forces are smaller than 0.05 eV/Å. To study the low doping concentration of N-doped ZrO_2_, a 96-atom 2*a* × 2*b* × 2*c* supercell (Zr_32_O_64_) is used for all the calculations.

The defect formation energy *E_f_*(*n*N_O_) of N-doped ZrO_2_ systems are calculated according to the following equation. 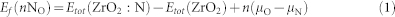
Here, *E_tot_*(ZrO_2_:N) is the total energy of the supercell with *n* oxygen atoms replaced by nitrogen atoms, and *E_tot_*(ZrO_2_) is the total energy of ZrO_2_ perfect crystal in the same supercell. *μ*_O_ is the chemical potential of oxygen (*μ*_O_ ≤ *μ*_O2_/2), and *μ*_N_ is the chemical potential of nitrogen in nitric oxide (NO)^48^.

For a supercell (Zr*_m_*O*_n_*N*_l_*) containing *m*Zr atoms, *n*O and *l*N atoms, the charge density difference is obtained as follows:
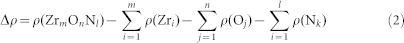
where, *ρ*(Zr*_m_*O*_n_*N*_l_*) is the charge density of supercell Zr*_m_*O*_n_*N*_l_*, *ρ*(Zr*_i_*), *ρ*(O*_j_*) and *ρ*(N*_k_*) are the charge density of isolated Zr*_m_*, O*_n_* and N*_k_* in the same supercell, respectively. According to the equation, the charge density difference gives a direct real-space visualization of the local charge redistribution due to the interactions of atoms.

To better understand the magnetic coupling of the Zr_32_O_62_N_2_ systems, SCD was used in all investigated systems and defined as:
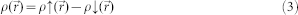
where 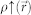 and 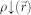 are the spin up and spin down charge densities of the Zr_32_O_62_N_2_ system, respectively.

## Author Contributions

H.Z. carried out calculations, J.L. and F.G. took analyses and discussion. H.Z. and J.L. wrote the manuscript. K.C., X.Y. and S.C. discussed and reviewed the manuscript.

## Supplementary Material

Supplementary Information Supplementary Information

## Figures and Tables

**Table 1:** Summary of the calculated results for all configurations of Zr_32_O_62_N_2_. The *d*_N-N_ is the N-N distances. The relative energy Δ*E* is referred to the (*a*, *b*) configuration and the most stable state (either FM or AFM) is used for each configuration. The magnetization energy Δ*E*_FM-AFM_ is the energy difference between the FM and AFM states, and *M* is the total magnetic moment for the FM state

Configuration (*i*, *j*)	*d*_N-N_ (Å)	Δ*E* (meV)	Δ*E*_FM-AFM_ (meV)	*M* (*μ*_B_)	*Coupling*
(*a, b*)	4.4	0	−372.7	2.0	FM
(*a, c*)	3.4	66.1	−356.3	2.0	FM
(*a, d*)	2.8	163.3	2.6	2.0	NM
(*d, e*)	2.6	286.9	−32.7	2.0	FM

**Table 2:** Atomic charge densities from Mülliken population analysis of Zr, O and N atoms

Atomic charge densities	Zr	O	N	
Zr_32_O_64_	1.47	−0.74	/	
Zr_32_O_63_N	1.46	−0.75	−0.65	
(*a, b*)	1.50	−0.75	−0.45(N3)	−0.88(N4)
(*d, e*)	1.5	−0.75	−0.65(N1)	−0.65(N2)
