# Supplementary material for: Nature of charge transport and p-electron ferromagnetism in nitrogen-doped ZrO2: An ab initio perspective
Source: Sci Rep. 2015 Feb 26;5:8586. doi: 10.1038/srep08586 (PMC4341218; doi:10.1038/srep08586)
Supplement: Supplementary Information [file srep08586-s1.doc]

*Supplementary Information*

**Nature of charge transport and *p*-electron ferromagnetism in nitrogen-doped ZrO_2_: An *ab initio* perspective**

Huanfeng Zhu^1^, Jing Li[[1]](#footnote-2)1,2,3, Kun Chen^1^, Xinyu Yi^1^, Shuai Cheng^1^, and Fuxi Gan^1,2^

^1^ Department of Optical Science and Engineering, Fudan University, Shanghai 200433, China

^2^ Shanghai Engineering Research Center of Ultra-Precision Optical Manufacturing, Fudan University, Shanghai 200433, China

^3^ Key Laboratory of Micro and Nano Photonic Structure (Ministry of Education), Fudan University, Shanghai 200433, China

***Figure Legends***

**Figure S1 The electronic band structure and the corresponding density of states for the pure 96-atoms 2×2×2 ZrO_2_ supercell.** The vertical dotted line indicates the Fermi level at 0 eV.

**Figure S2** **The isosurface SCD for ZrO_2_ with single N substituted at O3-type site.** The cyan, red and blue balls represent Zr, O and N atoms, respectively. The green (yellow) isosurface correspond to positive (negative) spin charge densities. The isovalue is set to 0.02 e/Å^3^.

**Figure S3 The charge density difference of ZrO_2_ with single N substituted at O3-type site.** The cyan, red and blue balls represent Zr, O and N atoms, respectively. The isovalue is set to 0.1 e/Å^3^.

**Figure S4 The schematic structure (a) and TDOS (b) of Zr_32_O_62_N_2_ with *d*_N-N_=7.4 Å.** The cyan, red and blue balls represent Zr, O and N atoms, respectively. The blue and red lines represent spin-up and spin-down states, respectively. The vertical dotted line indicates the Fermi level at 0 eV. The calculated results show that the TDOS is spin unpolarized, and some defect states are localized in the band gap.

**Figure S5 The charge density difference of (*a, b*) and (*d*, *e*) configurations in the FM states and the AFM states.** The cyan, red and blue balls represent Zr, O and N atoms, respectively. The isovalue is set to 0.1 e/Å^3^.

**Figure S1**


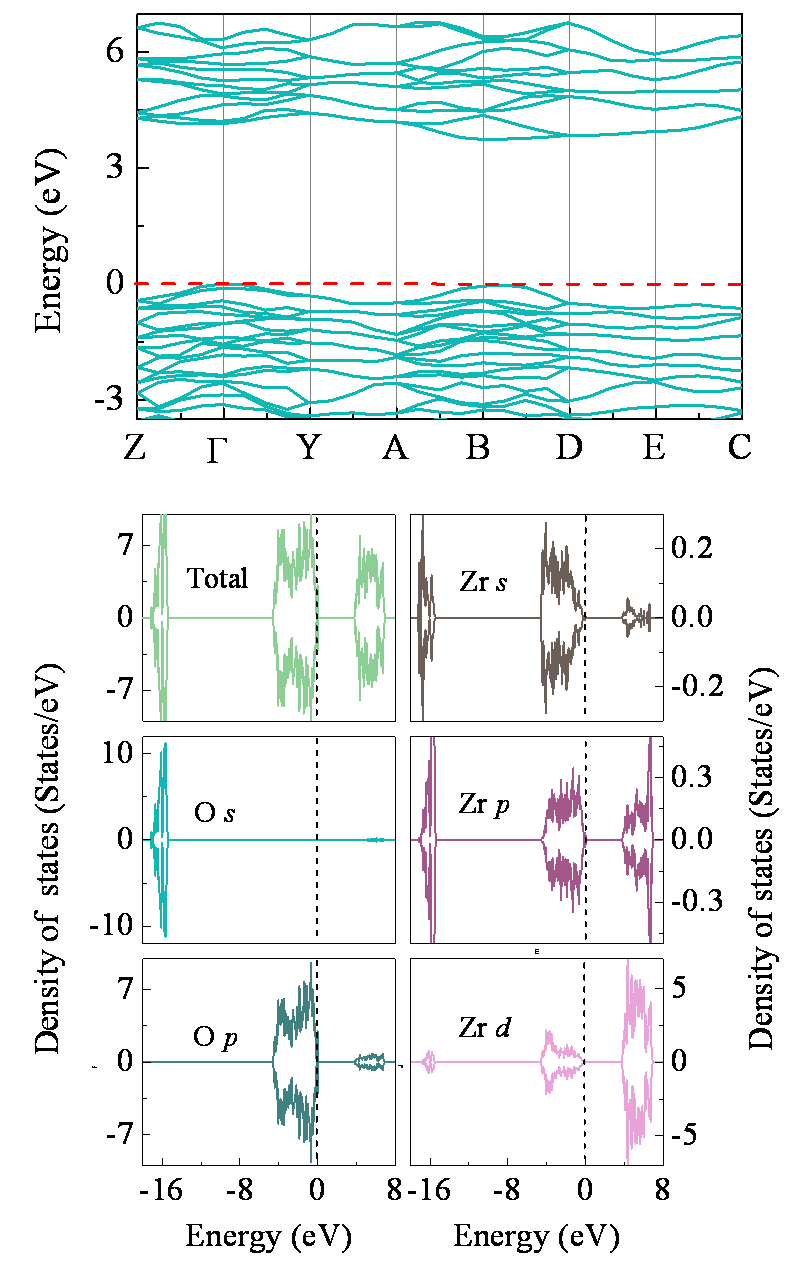


**Figure S2**


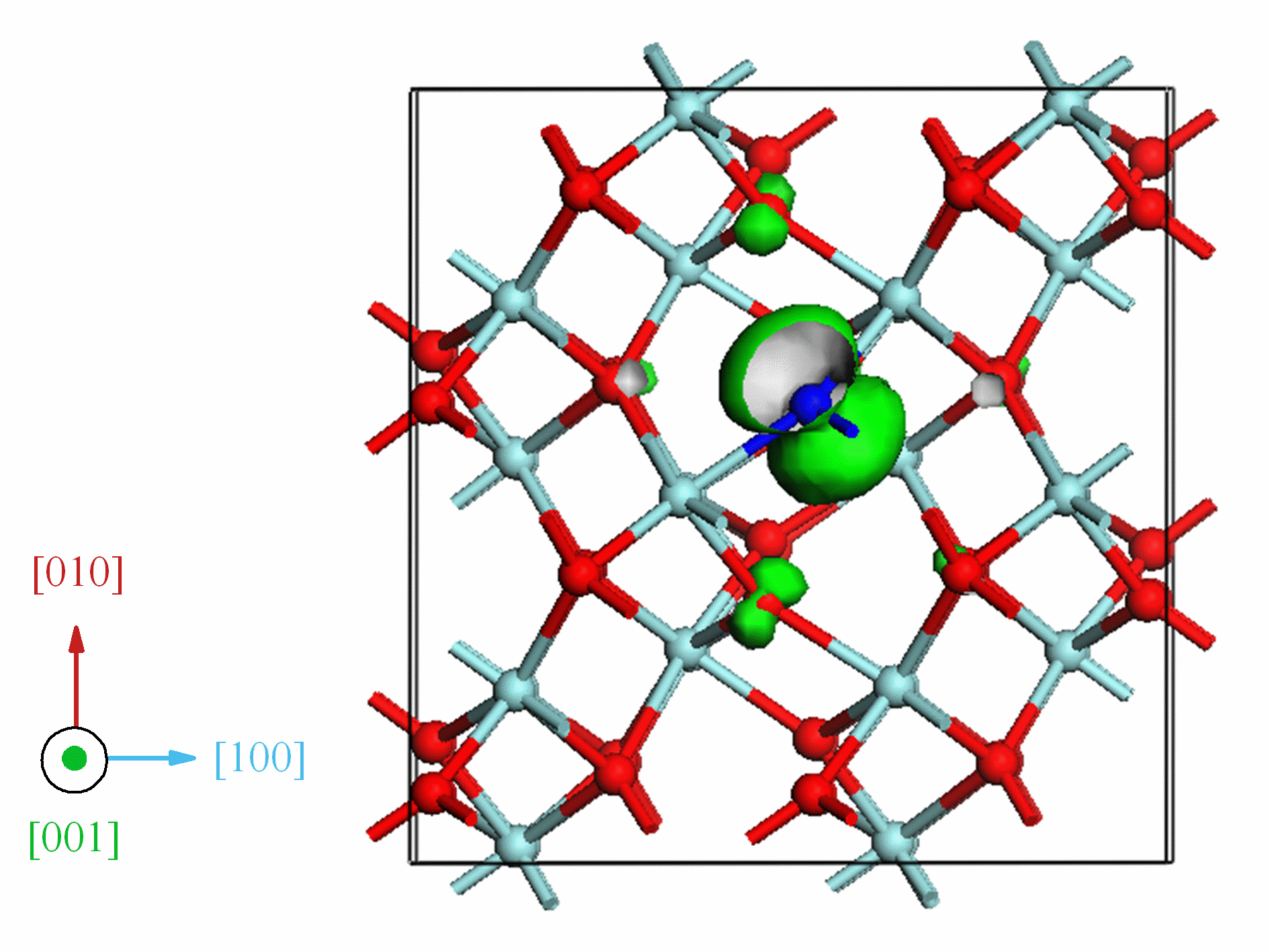


**Figure S3**


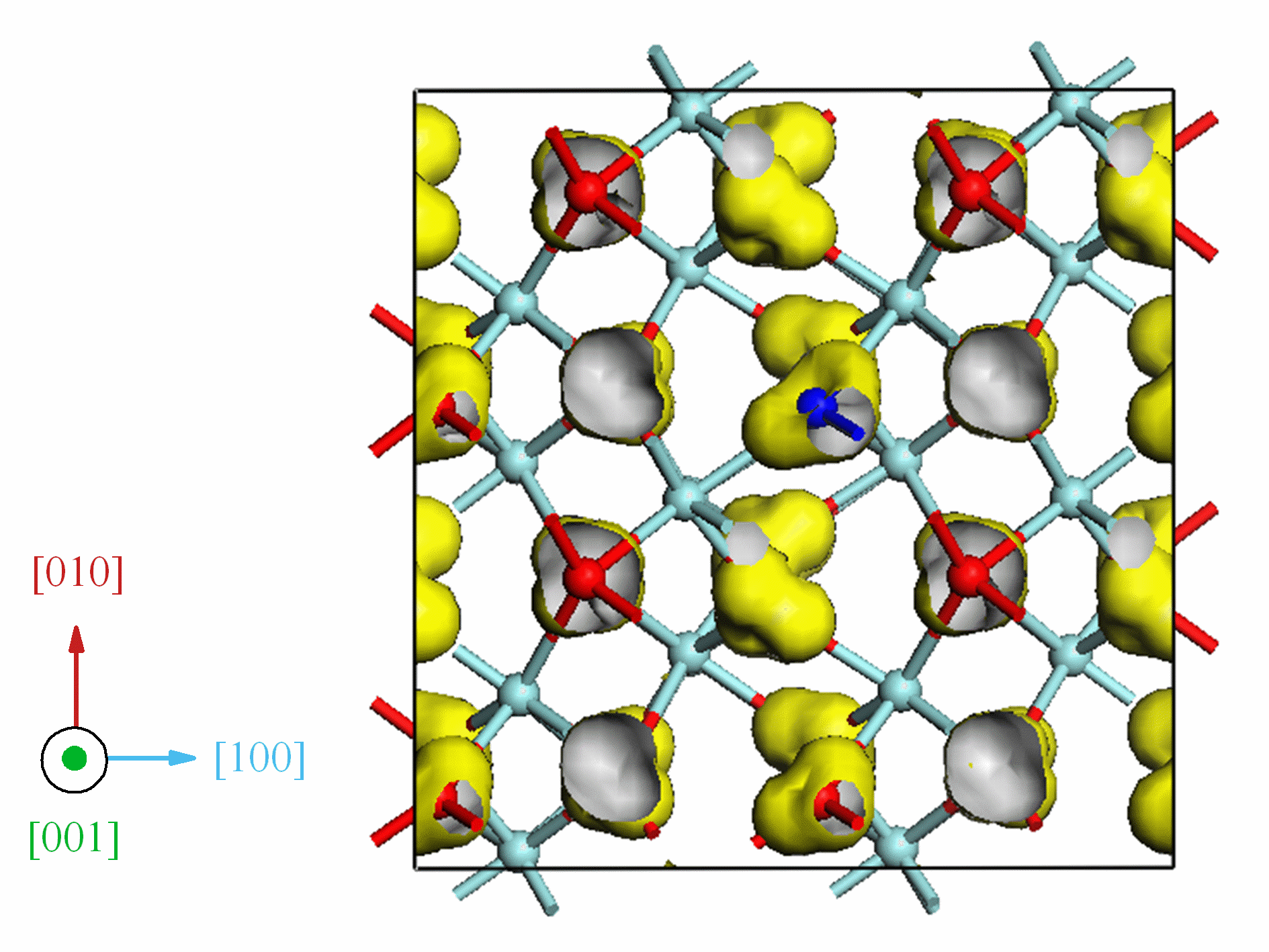


**Figure S4**


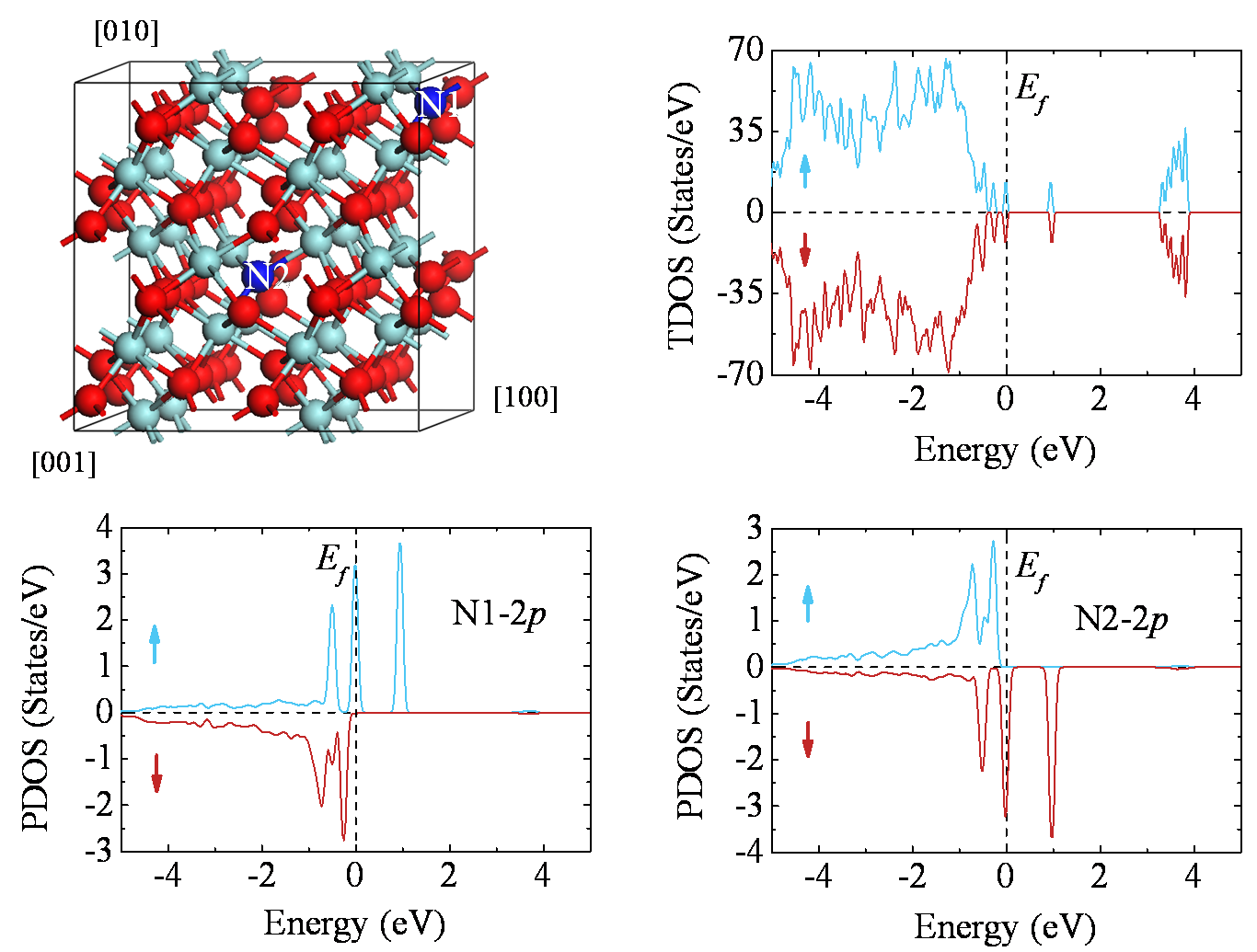


**Figure S5**


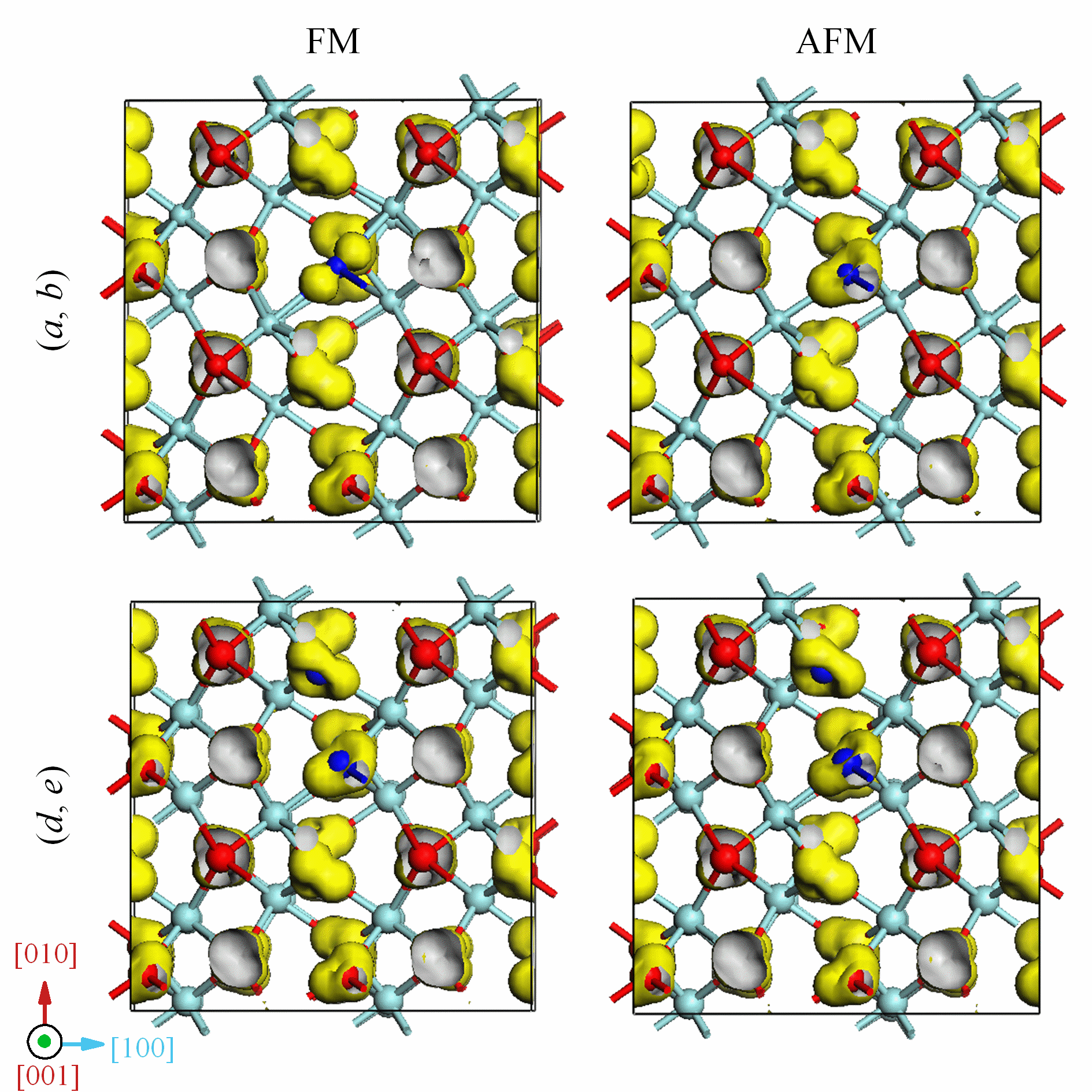


1. Email: [lijing@fudan.edu.cn](mailto:lijing@fudan.edu.cn) [↑](#footnote-ref-2)
